# Supplementary material for: Lysophosphatidylserine Induces MUC5AC Production via the Feedforward Regulation of the TACE-EGFR-ERK Pathway in Airway Epithelial Cells in a Receptor-Independent Manner
Source: Int J Mol Sci. 2022 Mar 31;23(7):3866. doi: 10.3390/ijms23073866 (PMC8999057; doi:10.3390/ijms23073866)
Supplement: Supplementary file 1 [file ijms-23-03866-s001.zip › ijms-1476437-supplementary.pdf]

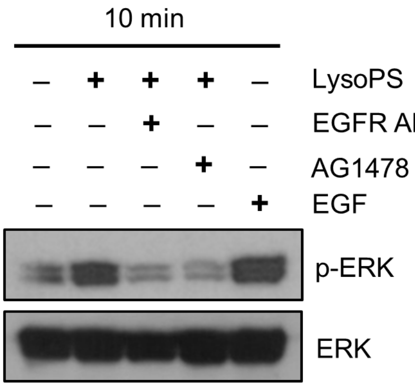

Supplementary Figure S1. EGFR neutralizing antibody inhibits LysoPS-induced ERK phosphorylation in the early phase as efficiently as the EGFR inhibitor AG1478. NCI-H292 cells were pretreated with EGFR neutralizing antibody (10 μg/mL) and AG1478 (10 μM) for 30 min, then treated with LysoPS and EGF for 10 min, and examined for ERK phosphorylation. Total ERK was used as a loading control.

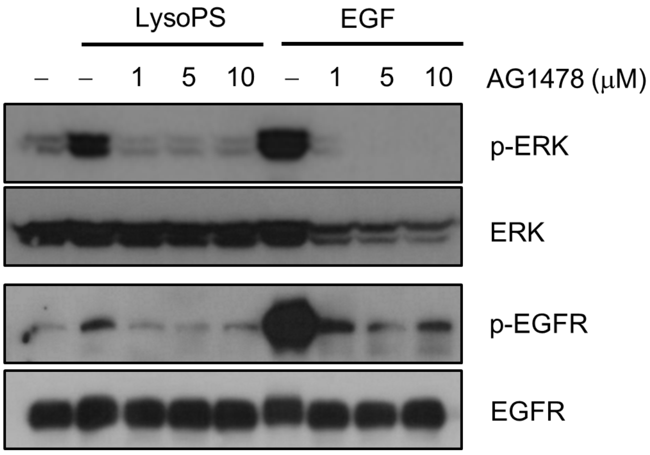

Supplementary Figure S2. LysoPS-induced ERK and ERGF phosphorylation were equally eliminated by the EGFR inhibitor AG1478. NCI-H292 cells were pretreated with different concentrations of AG1478, treated with LysoPS or EGF for 10 min, and examined for ERK and EGFR phosphorylation. Total ERK and EGFR were used as loading controls.
